# Pathological role of methionine in the initiation and progression of biliary atresia

**DOI:** 10.3389/fped.2023.1263836

**Published:** 2023-09-12

**Authors:** Zheng Jiachen, Tam Paul Kwong Hang, Wong Kenneth Kak Yuen, Lui Vincent Chi Hang

**Affiliations:** ^1^ Department of Surgery, School of Clinical Medicine, The University of Hong Kong, Hong Kong, Hong Kong SAR, China; ^2^ Faculty of Medicine, Macau University of Science and Technology, Macau, Macau SAR, China; ^3^ Department of Surgery, University of Hong Kong-Shenzhen Hospital, Shenzhen, China; ^4^ Dr. Li Dak-Sum Research Centre, The University of Hong Kong, Hong Kong, Hong Kong SAR, China

**Keywords:** biliary atresia, hepatotoxicity, hypermethioninemia, methionine metabolism, metabolic intermediates

## Abstract

Methionine (Met) is an essential amino acid, and its excessive dietary intake and/or its metabolism disturbance could lead to accumulation/depletion of hepatic Met and some of the key intermediates of these pathways, which would interfere normal liver function and would be associated with liver diseases. Biliary atresia (BA) is a life-threatening disease characterized by inflammatory fibrosclerosing changes of the intrahepatic and extrahepatic biliary systems and is the primary cause of obstructive neonatal cholestasis with a rapid course of liver failure. However, its pathogenesis remains unknown. Previous studies reported elevated Met level in patients with obstructive cholestasis, suggesting a potential link between Met and BA. This paper reviews the Met metabolism in normal conditions and its dysregulation under abnormal conditions, the possible causes of hypermethioninemia, and its connection to BA pathogenesis: Abnormal hepatic level of Met could lead to a perturbation of redox homeostasis and mitochondrial functions of hepatocytes, enhancement of viral infectivity, and dysregulation of innate and adaptative immune cells in response to infection/damage of the liver contributing to the initiation/progression of BA.

## 1. Methionine metabolism in the liver

**Figure 1:**
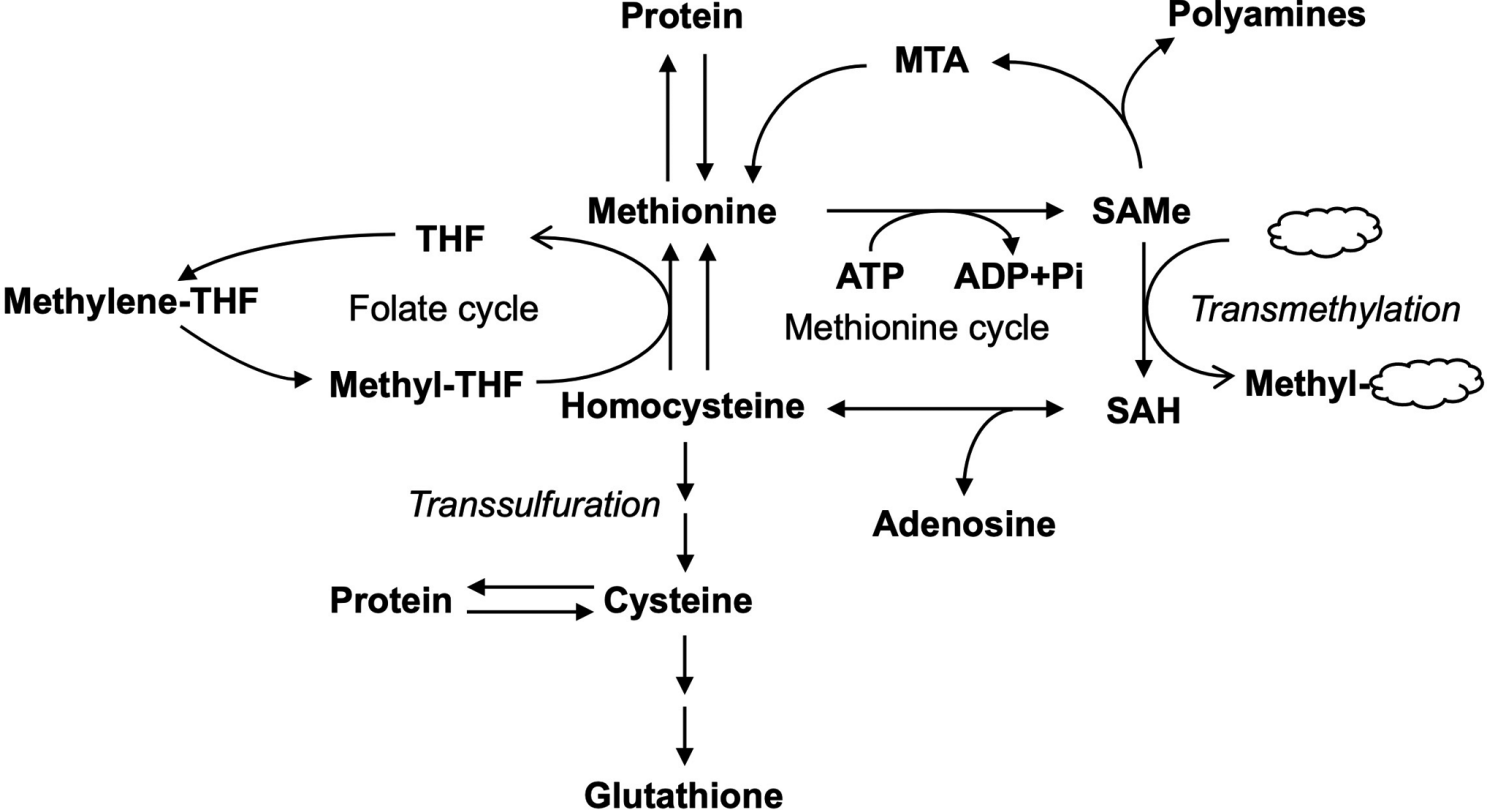
Metabolism of methionine in the liver. Methionine metabolism is linked to various metabolic pathways including folate cycle, transmethylation, and transsulfuration pathways. THF, tetrahydrofolate; MTA, methyl-thioadenosine; SAMe, S-adenosylmethionine; SAH, S-adenosylhomocysteine; ATP, adenosine triphosphate; ADP, adenosine diphosphate.

Methionine (Met) is an essential amino acid (AA), and nearly half of the Met consumed is metabolized in the liver in mammals. Met is first metabolized by methionine adenosyltransferase (MAT) to form S-adenosylmethionine (SAMe, which is also abbreviated as AdoMet) (1) (Figure 1). Two forms of MATs in mammals are identified: liver-speciﬁc MAT and non-liver-speciﬁc MAT. Both are products of two genes, *MAT1A* and *MAT2A*, respectively. *MAT1A* encodes two liver-specific MAT isoenzymes, such as MATI (MAT1A homo-tetramer) and MATIII (MAT1A homo-dimer), that catalyze the transfer of an adenosyl group of adenosine triphosphate (ATP) to Met to produce SAMe and tripolyphosphate (1, 2). A third isoenzyme, MATII, is encoded by the second gene, *MAT2A*, and is expressed in all tissues, such as the liver, albeit to a smaller extent (2).

SAMe serves mainly as a methyl donor in the cell and transfers its methyl group to substrates such as nucleic acids, proteins, lipids, and secondary metabolites with the formation of S-adenosylhomocysteine (SAH, which is also abbreviated as AdoHcy), via transmethylation (3, 4). SAH is subsequently hydrolyzed by SAH hydrolase to form homocysteine (Hcy) (5). Hcy can either be remethylated via the transfer of the methyl group from methyltetrahydrofolate (methyl-THF) with the formation of tetrahydrofolate (THF) in the folate cycle to regenerate Met or enter the transsulfuration pathway forming other products such as cysteine and the important antioxidant glutathione (GSH) (6). SAMe can also regenerate Met via methyl-thioadenosine (MTA), a byproduct of polyamines synthesis. SAMe-dependent methylation is central to many biological processes. Up to 85% of all methylation reactions occur in the liver (7).

## 2. Regulation of Met metabolism in the liver

**Figure 2:**
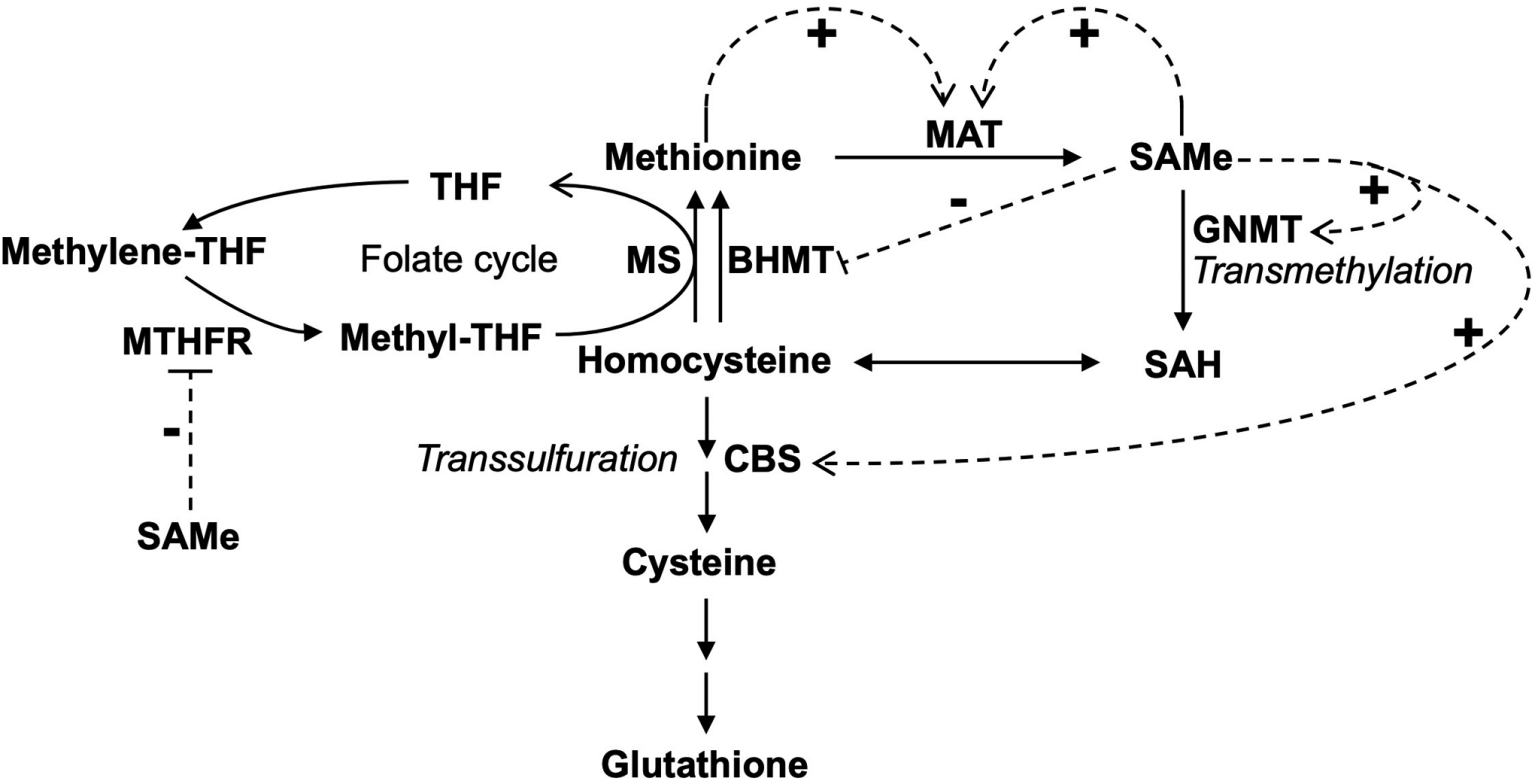
Feedback regulation of methionine metabolism in the liver. Feedback activation and inhibition of enzymes by methionine and SAMe were shown. The dotted arrow with a “+” sign indicates allosteric activation. The dotted line with a “−” sign indicates allosteric inhibition. MAT, methionine adenosyltransferase; SAMe, S-adenosylmethionine; SAH, S-adenosylhomocysteine; CBS, cystathionine-β-synthase; GNMT, glycine N-methyltransferase; THF, tetrahydrofolate; MTHFR, methylene-THF reductase; MS, methionine synthase; BHMT, betaine–homocysteine methyltransferase.

**Figure 3:**
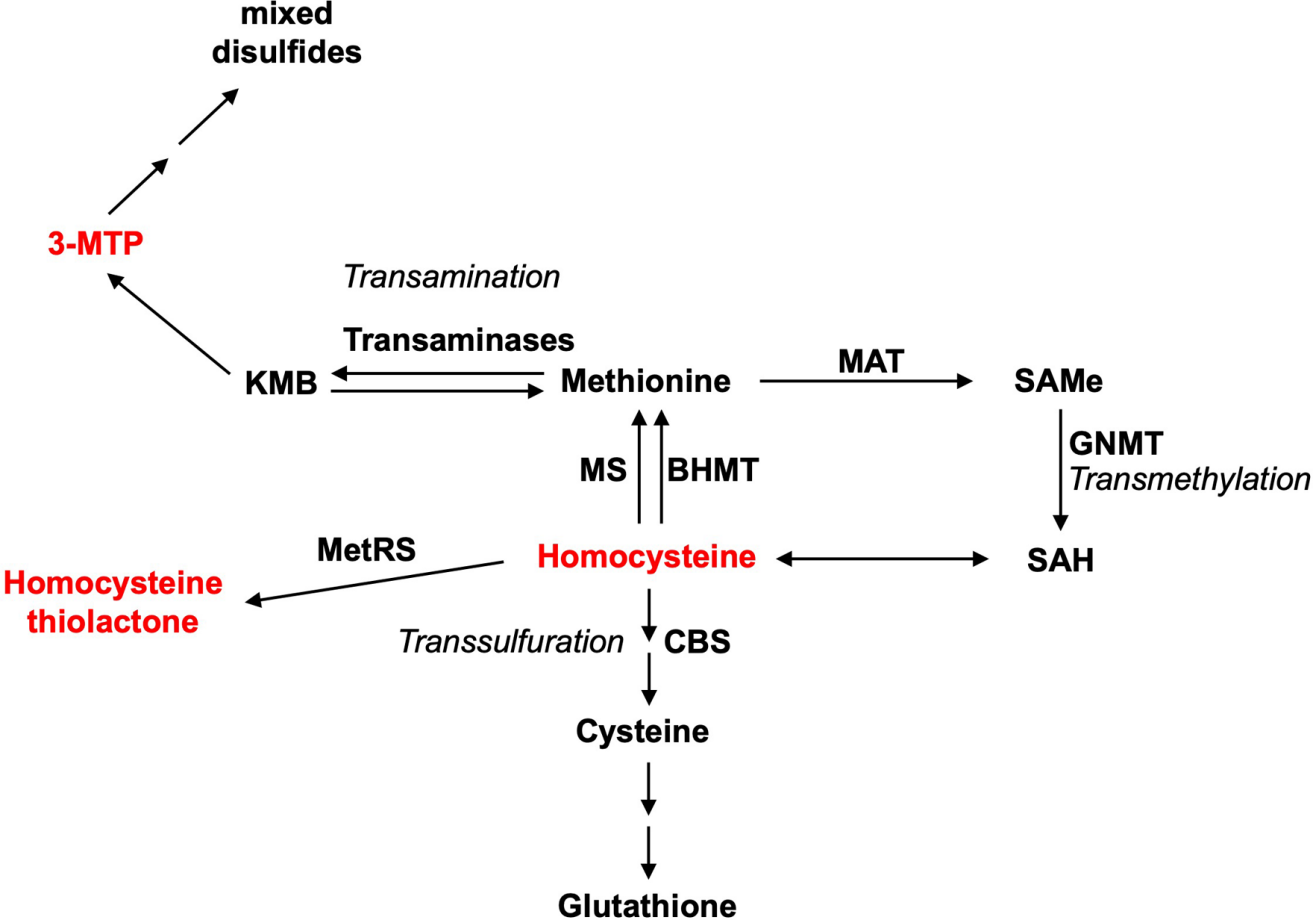
Some metabolic intermediates of methionine are toxic. Three main pathways for methionine metabolism are as follows: transamination, transmethylation, and transsulfuration. Methionine metabolism is a pivot linking these pathways. The toxic metabolite intermediates of methionine metabolism are indicated in red. MAT, methionine adenosyltransferase; SAMe, S-adenosylmethionine; SAH, S-adenosylhomocysteine; CBS, cystathionine-β-synthase; GNMT, glycine N-methyltransferase; MS, methionine synthase; BHMT, betaine–homocysteine methyltransferase; 3-MTP, 3-methylthiopropionic acid.

In the liver, when the consumption of Met exceeds its average dietary intake, *de novo* synthesis of Met occurs via the transfer of a methyl group from methylene-THF first by methylene-THF reductase (MTHFR) and then by Met synthase (MS) to Hcy to form Met to make up the difference (8, 9) (Figure 2). SAMe is an allosteric activator of cystathionine-β-synthase (CBS) (10–12), glycine N-methyltransferase (GNMT) (13), and MAT (14–16). In contrast, it is an allosteric inhibitor of MTHFR (17). Furthermore, SAMe stabilizes CBS against proteolytic degradation (18), upregulates *MAT1A* expression (19), and inhibits the expression of betaine–homocysteine methyltransferase (BHMT) (20). Low Met levels lead to a decrease of hepatic SAMe, releasing its inhibition on BHMT and MTHFR, which promotes the synthesis of Met to restore normal levels of Met. On the contrary, high Met levels result in elevated hepatic SAMe, which in turn leads to the activation of Met catabolism (via the transmethylation and transsulfuration pathways) and the inhibition of Met regeneration, thus restoring normal levels of Met (Figure 3). In sum, SAMe is a key regulator of Met metabolism, and Met concentration is closely related to the level of SAMe (21, 22).

Met metabolism is closely linked to various metabolic pathways such as folate cycle and transsulfuration pathway (23, 24). Hence, abnormal dietary intake of Met and/or dysregulation of Met metabolism could lead to accumulation/depletion of hepatic Met and some of the key intermediates of these pathways, which could be harmful to the liver and associated with liver diseases.

## 3. Biliary atresia

Cholestasis is the failure of bile from the liver to reach the duodenum, and neonatal cholestasis (NC) is defined as the persistence of a direct bilirubin concentration of >20% of the total serum bilirubin of newborns for more than 14 days after birth. NC is attributed to a wide range of conditions including obstructive, infectious, metabolic, and genetic causes with variable incidence.

Biliary atresia (BA) is a life-threatening disease characterized by inflammatory fibrosclerosing changes of the intrahepatic and extrahepatic biliary systems that shuts down the bile outflow from the liver and is the primary cause of obstructive NC. The incidence of BA is 5–20 per 100,000 newborns, with the highest incidence among Asians, and its pathogenesis remains unknown. If left untreated, within 2 years, this disease would progress to biliary fibrosis and eventual hepatic failure requiring liver transplantation. A procedure called Kasai hepatoportoenterostomy (KPE) that replaces the obliterated extrahepatic bile duct with an intestinal conduit is the first-line treatment to re-establish the bile flow with only 50% success rate (25). As intrahepatic cholangiopathy remains unresolved by surgical intervention, patients develop cholangitis (50%), portal hypertension (60%), and progressive fibrosis, leading to liver failure (50%) requiring liver transplantation with lifelong immunosuppressants. In fact, BA is the most common reason for pediatric liver transplantation.

BA is most likely a clinical phenotype resulting from a number of prenatal or perinatal insults to the hepatobiliary tree, and this disease is underpinned by a number of key pathobiological factors (KPFs) either singly or more often in combinations. These KPFs include genetic predisposition and injury triggered by viral infections/toxins and/or niche abnormalities, with inflammatory and immune dysfunction, leading to obliteration and eventual fibrosis and atresia of the bile ducts (26).

## 4. Hypermethioninemia-induced liver damage

### 4.1. Possible causes of hypermethioninemia

Hypermethioninemia (plasma concentration of Met above 45μM) (27) could be induced by a number of causes such as high Met diet, dysregulation of Met metabolism, or genetic causes including MATI/MATIII deficiency, CBS deficiency, and GNMT deficiency (28). An elevated Met level is hepatotoxic (29–32).

BA patients’ diet primarily consists of breast milk and milk powder. Hence, their mother's high Met diet could increase the Met level in the breast milk, which subsequently causes a passive intake of excessive Met in their babies. In addition, as one of the essential amino acids, Met supplement in milk powder is accepted. However, the concentration of Met in the milk powder varies among brands. Some brands offer milk powder with high Met content.

### 4.2. Evidence supporting the link between hypermethioninemia and liver injury

In rabbit, intravenous administration of Met (121 mg/kg/d) could lead to a blockage of bile flow and jaundice (33), and these liver damages are similar to parenteral nutrition cholestasis (33). Furthermore, hypermethioninemia induces oxidative damage and liver injury (34), hemolytic anemia, and development of its associated symptoms such as jaundice and enlarged liver and spleen in rats (35–37). In addition to inducing cholestasis, hypermethioninemia may induce inflammation, raise cytokine production, and therefore impair the hepatocellular transport systems that mediate biliary excretion of bile (38). Increased inflammatory cells, especially in the portal space connective tissues, were reported in the rats that were fed with a high Met diet (34). The Met level is markedly elevated in patients with cholestasis, especially with obstructive jaundice, which indicates the potential association between Met and BA (39–41). The following sections aim to review the existing findings on the effects of the elevated level of Met on cellular functions, immune system, viral infectivity, and liver fibrosis and on how the hepatic Met level contributes to the disease initiation/progression of BA.

### 4.3. Metabolic intermediates of methionine

Met transmethylation begins with the ATP-dependent conversion of Met to SAMe (1). ATP depletion from excessive SAMe formation and the accumulation of SAMe itself have been correlated with hypermethioninemia-induced hepatotoxicity (30, 42). Further, accumulation of SAMe could lead to an elevated level of Hcy, which can then be catalyzed by methionyl–tRNA synthetase (MetRS) to form lysine-reactive metabolite Hcy thiolactone (1). Hcy and Hcy thiolactone promote arteriosclerosis via increased oxidant stress, impaired endothelial function, and induction of thrombosis, increasing the cardiovascular disease risk in humans (43). However, the link between Hcy and Hcy thiolactone in Met-induced liver toxicity has yet to be demonstrated. Met transamination produces 2-keto-4-methylthiobutyric acid (KMB) (44, 45), which is then oxidatively decarboxylated to form 3-methylthiopropionic acid (3-MTP) (46, 47). 3-MTP is further converted into highly toxic and volatile molecules such as methanethiol, hydrogen sulfide, and dimethylsulfide (46, 48–50). Methanethiol has been shown to inhibit enzymes that protect against peroxidative damage similar to the elevated level of Met (51). Similar to chow spiked with Met, 3-MTP-spiked chow induced the same toxicological symptoms of growth retardation and hemolytic anemia in rats (46).

## 5. Disturbance of cellular redox homeostasis

Cellular redox homeostasis is an essential and dynamic process that ensures the balance between reducing and oxidizing reactions within cells and is critical for normal cellular functions. Proteins, lipids, and DNA of hepatocytes are among the cellular structures that are primarily affected by the oxidant and antioxidant imbalance within the cell, which participates in the course of inflammatory, metabolic, and proliferative liver diseases. The cellular redox homeostasis is substantiated primarily by GSH, which represents the major redox buffer in the maintenance of cellular redox homeostasis (52).

Met is a substrate for GSH, and intake of dietary Met directly affects the hepatic GSH level. High Met diet causes alteration of hepatic prooxidant/antioxidant status and oxidative stress parameters in rats (29, 34). Hypermethioninemia could lead to the upregulation of the transamination pathway, 3-MTP would be increased, and its metabolites would inhibit the enzymes involved in resisting peroxidative damage. Incubation of mouse primary hepatocytes in Met or 3-MTP induced a decrease of the GSH level and cellular damage (53). Met and its derived sulfur metabolites in the transamination pathway could also activate histone acetylase general control non-depressible 5 (GCN5) acetyltransferase, promoting acetylation of the transcriptional coactivator PGC-1α to suppress hepatic gluconeogenesis (54). Since glucose could produce intracellular GSH via the pentose phosphate pathway, suppression of gluconeogenesis could result in lower GSH levels (55).

Met is directly converted by MATI/MATIII into SAMe, and MATI/MATIII deficiency could lead to decreased SAMe levels and its downstream products including GSH. Bile duct ligation (BDL) caused obstructive jaundice, abnormal liver function, increased lipid peroxide levels, and decreased GSH levels in rats, which indicated that oxidative stress in rats with obstructive jaundice was observed. SAMe application alleviated these injuries in rats that underwent BDL (56). Deletion of *Mat1a* in mice resulted in depleted liver SAMe levels and reduced GSH levels, hypermethioninemia, marked changes in the expression of many enzymes of Met metabolism, and increased expression of many acute-phase markers and growth-related genes (57). A high level of hepatic Met lowered the hepatic gluconeogenesis via transamination and could contribute to the progress of liver injury (54). Reduced Mat1a function is strongly associated with metabolic disorders, in particular fatty liver disease characterized by lipid accumulation and immune dysfunction, which rendered *Mat1a*-*/*- mice more susceptible to liver injury (57). In cirrhotic liver, MAT1A expression is markedly diminished (58), which resulted in a low SAMe level and a low GSH level. Exogenous SAMe supplement increased the GSH level in the liver and alleviated the liver damage (7, 56, 59). Mitochondrial polarization was also seen restored in MAT1A-KO hepatocytes upon incubation with SAMe (60).

Mitochondria, as an “energy factory” in eukaryotic cells, are the primary source of reactive oxygen species (ROS) in hypoxic cells and participate in the regulation of redox homeostasis. The establishment of the mitochondrial membrane potential is an essential component in the generation of ATP during oxidative phosphorylation. Disruption of the mitochondrial function and mitochondrial membrane potential is associated with the pathophysiology of liver diseases (61). Hepatocytes in *Mat1a-/-* mice showed a higher expression of cytochrome P450 family 2 subfamily E member 1 (CYP2E1) and a reduction in mitochondrial membrane potential (62–64). The influence of MAT1Aon the mitochondrial function is largely mediated by its direct methylation regulation on CYP2E1 (64). A high level of CYP2E1 activity could disrupt the mitochondrial functions and mitochondrial membrane potential and increase the production of ROS, promoting the progression of liver damages in *Mat1a-/-* mice (65, 66). Methionine metabolism disorder can aggravate the damage in the pathological state of a disease. A high methionine concentration can cause oxidative stress of liver cells, which could induce cell death causing liver injury. Further studies need to investigate whether hypermethioninemia-induced oxidative stress of liver cells is involved in the initiation and/or disease progression of BA.

## 6. Enhancement of virus infectivity

Oxidative stress exacerbates the pathogenesis of coxsackievirus B3 (CVB3) infection in mice, and the change in virulence is attributed to changes in the viral genome and in the immune functions of the oxidatively stressed mice (67). Depletion of GSH is observed in patients with hepatitis virus, HIV, HSV-1, etc. (68–70). GSH may influence the viral infection by the (a) regulation of nuclear factor kappa B (NF-kappa B) activation of the infected cells (71), (b) interference of virus entry (72), and (c) inhibition of apoptosis and the release of active virus from the infected cells (73).

Perinatal cytomegalovirus (CMV) infection has been suggested to be a possible cause or trigger of BA, in that CMV infection initiates damage to the bile duct, which is then followed by autoimmune responses targeting the bile duct. It was found that approximately 10% of BA patients showed serum CMV IgM+, and CMV IgM + BA patients usually have a worse outcome with reduced clearance of jaundice, native liver survival, and increased mortality, representing a distinct clinical and pathological entity of BA with a diminished response to KPE (74, 75).

In mammals, DNA methyltransferases (DNMTs), such as DNMT1, DNMT3A, and DNMT3B, write and regulate the DNA methylation patterns, which in turn regulate gene expression. DNMT1 and DNMT3b cooperate to silence genes in human cancer cells, and deletion of *DNMT1* and *DNMT3b* eliminates methyltransferase activity and reduces genomic DNA methylation (global DNA hypomethylation) by greater than 95% (76). Interestingly, CMV infectivity of cells is greatly influenced by the methylation status of the cells. Deletion of *DNMT1* and *DNMT3b* either alone or in combination significantly reduces the genomic methylation but increases CMV infectivity of the host cells (77). The enhancement of viral infectivity could be attributed to the hypomethylation of STAT1, leading to a reduced binding of STAT1 to the interferon-stimulated response element and thus reduction of the expression of interferon-stimulated genes (ISGs), weakening the antiviral effects of interferon (78, 79). Convallatoxin could inhibit the CMV infection and replication by reducing the cellular Met import (80). Bile acids exert anti-CMV effects by suppressing the CMV-induced gene expression and diminishing the virus production in hepatocytes (81). The intake level of dietary Met could affect the lipid and bile acid metabolism in the liver in mice, and Met-restricted diet could restore the normal bile acid pool in the liver (82). Therefore, the intake level of dietary Met may indirectly affect the CMV infectivity of hepatocytes.

## 7. Disruption of the immune system

SAMe increases the suppression competency of regulatory T cells (Treg cells) in a dose-dependent manner via affecting the expression of FOXP3 (83), promoting the proliferation of CD8+cytotoxic T cells. Methionine also functions as a key nutrient affecting epigenetic reprogramming in CD4+ T helper (Th) cells. Th cells are central drivers of autoimmune pathology, and methionine restriction could limit the expansion of inflammatory Th17 cells (84). High concentrations of Met are needed to activate T cells and maintain their activation status (85), which indicates that hypermethioninemia promotes T-cell activation. Elevated levels of MTA and SAMe are tightly linked to T-cell exhaustion in hepatocellular carcinoma (HCC), and deletion of a key SAMe-producing enzyme MAT2A results in inhibition of T-cell exhaustion and limitation of HCC growth in mice (86). It was suggested that SAMe or MTA inhibited effector T-cell function after activation, driving them to an exhausted post-activation state via the regulation of the global methylation and chromatin accessibilities of T cells.

Kupffer cells (KCs), which are the largest population of resident macrophages in the liver, are the first innate immune cells and protect the liver from infections. Upon activation, cytokine and chemokine production by activated KCs is involved in the pathogenesis of liver damage. A lower SAMe level could attenuate the suppressing activation of the TLR4/MAPK pathway, therefore upregulating the tumor necrosis factor-alpha (TNF-α) expression in KCs (87). Met could also attenuate lipopolysaccharide-induced inflammation in macrophages via inactivation of the MAPK pathway and alteration of DNA methylation (88).

## 8. Accelerating the course of liver fibrosis

Hepatic stellate cells (HSCs) are liver-specific mesenchymal cells that retain features of resident fibroblasts. They are located in the space of Disse and maintain close interactions with sinusoidal endothelial cells and hepatocytes. In chronic liver damage, HSCs transdifferentiate from a “quiescent” to an “activated” state and are responsible for collagen deposition in the liver tissue, playing a key role in the fibrosis process. Via the activation of TLR4/MAPK signaling in KCs (87), low SAMe levels induce the expression of transforming growth factor-β (TGF-β) in KCs (89), which in turn leads to the activation of HSCs.

In addition to HSCs, in an injured liver, hepatocytes and bile duct cells can also transform into fibroblasts via the process of epithelial–mesenchymal transition (EMT), and excessive extracellular matrix (ECM) deposition by fibroblasts contributes to liver fibrosis. Under the stress of liver injury, bile duct cells undergo EMT and express fibroblast markers such as fibroblast-specific protein 1 (FSP-1), vimentin, and other mesenchymal markers in a diseased liver with ductular proliferation (90). Bile duct cells express a number of cytokines and pro-fibrogenic growth factors such as TGF-β1 and TGF-β2, which promote fibrosis of the liver parenchymal cells in an injured liver (91, 92). Blocking EMT of bile duct cells alleviated bile duct ligation, inducing ductular reaction and biliary fibrosis in mice (93).

Zeisberg et al. first reported the dedifferentiation of hepatocytes into fibroblasts via EMT (94). In the fetal liver, hepatocytes express both hepatic and mesenchymal markers, which indicates that fetal hepatocytes exhibit both hepatocytic and fibroblastic characteristics (95, 96). Hepatocyte nuclear factor 4-alpha (HNF4α), a transcription factor, controls the expression of hepatic genes and regulates the development of hepatocytes. HNF4α, in cooperation with its target HNF1α, directly inhibits transcription of the EMT master regulatory genes, *Snail* and *Slug*, repressing the mesenchymal program and EMT of hepatocytes (97). Knockdown of HNF4α in HNF4α-positive epithelial liver cancer cells promotes EMT and induces cell migration (98). Expression of HNF4α at the protein and transcript levels decreases in many liver diseases (99). All these suggest that hepatocytes may downregulate the expression of HNF4α in an injured liver, which promote the EMT of hepatocytes and contribute to the fibrosis process (100).

Hepatocytes could also dedifferentiate into hepatic progenitor cells (HPCs, which are identified by markers such as SMA, SOX9, KRT19, EpCAM, and PROM1), and the proliferation and differentiation of hepatocyte-derived HPCs are closely associated with ductular reactions and portal fibrosis in liver injury (101). HPCs isolated from *Mat1a*-*/*- mice transformed into fibroblastic cells when incubated with FGF10 and TGF-β1, and these HPC-derived fibroblasts were significantly expanded in RRV-induced BA mouse model (102). TGF-β1 could also induce adult mouse hepatocytes to undergo EMT, and up to 45% of the FSP1-postive fibroblasts are derived from hepatocytes in carbon tetrachloride (CCL4)-induced fibrotic liver (94).

## 9. Deletion of *Mat1a* promotes liver injury

Deletion of *Mat1a* enhances the susceptibility to liver injury in mice (57). Spontaneous macrovesicular steatosis and predominantly periportal mononuclear cell infiltration were observed in *Mat1a*-*/*- mice (57). Meanwhile, lipid metabolism-related genes were upregulated with increased hepatic triglyceride levels (62), which could be reversed by Met restriction (103). High Met diet could also stimulate cholesterol synthesis and promote the accumulation of hepatic total lipids (104). The cholesterol and lipid accumulation in hepatocytes could induce malfunction of hepatocytes.

**Figure 4:**
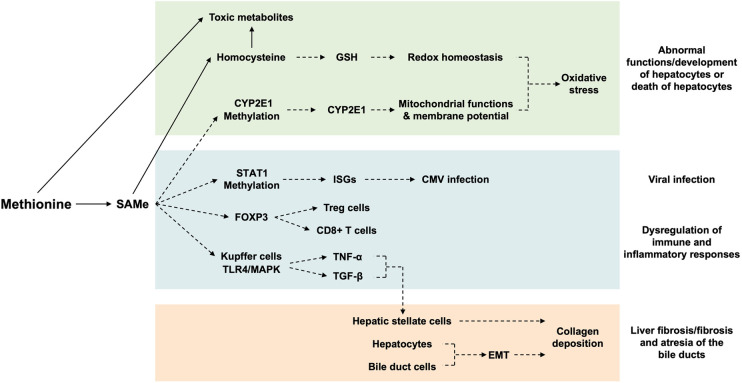
Mechanisms of actions of hepatic methionine on the liver. The hepatic level of Met affects the (i) hepatocyte development/functions and survival via disturbing the redox homeostasis/mitochondrial function/membrane potential of hepatocytes, (ii) CMV infectivity and immune/inflammatory responses to infection/injury of the liver via regulating the expression of genes relevant to CMV infection/T-cell development/cytokine production of immune cells, and (iii) process of fibrosis via activation of hepatic stellate cells and EMT of hepatocytes/bile duct cells. Arrows indicate metabolism; broken arrows indicate the direction of action. SAMe, S-adenosylmethionine; GSH, glutathione; CYP2E1, cytochrome P450 family 2 subfamily E member 1; STAT1, signal transducer and activator of transcription 1; ISGs, interferon-stimulated genes; CMV, cytomegalovirus; FOXP3, forkhead box P3; TLR4, Toll-like receptor 4; MAPK, mitogen-activated protein kinase; TNF-α, tumor necrosis factor-alpha; TGF-β, transforming growth factor-beta; EMT, epithelia–mesenchymal transition.

All of the above suggest that abnormal dietary intake and defective metabolism of Met could result in the abnormal levels of Met and its metabolites (SAMe and GSH) and accumulation of toxic metabolic intermediates (Hcy, Hcythiolactone, and 3-MTP) in the liver, which in turn affects the functions and the inflammatory responses to damage/infection of the liver (Figure 4).

## 10. Conclusion

In sum, perturbation of Met metabolism may promote the damage process of hepatobiliary injury triggered by viral infections/toxins and/or niche abnormalities, with dysregulated inflammatory and immune responses of the liver, and contribute to the obliteration and eventual fibrosis and atresia of the bile ducts in the initiation/progression of BA.
